# Towards a phronetic space for responsible research (and innovation)

**DOI:** 10.1186/s40504-016-0040-8

**Published:** 2016-05-20

**Authors:** Emanuele Bardone, Marianne Lind

**Affiliations:** Institute of Education, University of Tartu, Tartu, Estonia

**Keywords:** Phronesis, Responsible research and innovation, Technocratism, Science governance, Responsibility, Care, Inner goods, Tinkering

## Abstract

The term Responsible Research and Innovation has recently gained currency, as it has been designated to be a key-term in the European research framework Horizon 2020. At the level of European research policy, Responsible Research and Innovation can be viewed as an attempt to reach a broader vision of research and innovation as a public good. The current academic debate may be fairly enriched by considering the role that phronesis may have for RRI. Specifically, in this paper we argue that the current debate might be fruitfully enriched by making a categorial shift. Such a categorial shift involves moving away from the temptation to interpret responsible research and innovation in a technocratic way towards a more pluralistic vision that is rooted in the idea of phronesis. In the present context phronesis points the attention to the cultivation and nurturement of the researcher’s formation as a type of engagement with the actual practice of researching, a practice in which researchers (and other parties concerned) are called to apply judgment and exercise discretion in specific and often unique situations without the re-assuring viewpoint of the technician.

## Conceptions of intervention and models of deliberation

The first element that we are going to treat comparatively is intervention. Responsible Research and Innovation as a proposal concerning science governance is indeed a form of intervention. That is, RRI is not merely describing a state of affairs. Conversely, it is supposed to inform the way in which we, as a society, can realistically intervene so as to steer research and innovation towards our *desiderata* avoiding at the same time the negative consequences. In von Schomberg’s words, Responsible Research and Innovation is “a [a]design strategy which drives innovation and gives some ’steer’ towards achieving societal desirable goals” (Schomberg 2013, p. 48).

Specifically, the main aim of this first section is to show that our interpretations of what intervention is may vary significantly depending on the specific approach we take. In the second part of the section we will also show how we may derive a particular conception of deliberation in relation to the way in which intervention is interpreted. We will start from the technocratic approach.

### The technocratic approach: intervention as fabrication

The main element characterising the technocratic approach is fairly well summarised by Jullien. In the technocratic approach:

we set up an ideal form (eidos), which we take to be a goal (telos), and we then act in such a way as to make it become fact. (Jullien 2004, p. 3)

In this context intervening means bringing about directly and purposefully a specific outcome that is desired and/or expected. In this sense, intervention can be interpreted as a way of *making* or a form of *fabrication*, as it aims to produce something specific in the world. Intervention has some fundamental similarities with, for instance, building a bike frame, a house, a bridge, etc. When we make or build something we usually start off from an ideal form. That is, something that is essentially a model, a representation of that which we want to realise. We then subsequently act so as to make it real the same way as we build a house or a bridge by following a plan or a model, which directly *and* purposefully gives shapes to the way in which we should proceed.

It follows that the idea of deliberation that is more or less tacitly assumed is the one in which we start off from the goal (or end) that we want to achieve and we subsequently walk *backwards* to identify the most effective means, that is, the steps that we need to take in order to bring about the desired goal. This implies that deliberation can be turned into an object of objective analysis. That is meant to generate *predictive knowledge*, that is, knowledge that serves the purpose of predicting the causal factors at play in the means-end chain and thus bringing them under our control (Dunne 1993). The predictive knowledge can be codified in a technique (or a set of techniques) informing how to intervene. Such a technique can then be used and re-used effectively like a kind of recipe bringing us step by step closer to the desired outcome.

Interestingly, this type of knowledge is practical in the sense that it concerns the production of “outcomes”. Yet it is fundamentally theoretical in the sense that it is derived from a model or a plan, that is, an idea. It follows that the limits and the potential drawbacks we may suffer from derive from the limits of our technique, which ultimately concerns the identification of the causal factors at play in a given domain. The scientific enterprise has therefore the role of trying to catch up with our ignorance about those causal factors. In the technocratic approach, those who possess the knowledge to make something are then identified as experts. The experts are those who have the kind of knowledge about the cause-effect relationships guiding one’s own actions and strategies.

Overall, the technocratic model of deliberation is characterised by what is usually called in cognitive psychology “tunnel vision” (Williams 1988). That is, a very strict criterion of relevance in choosing between different options of action is adopted, which is always established beforehand. In doing so, any occurrences or happenings falling outside the predetermined path are inevitably filtered out. In this sense nothing can be left to chance, as chance is essentially viewed as a potential obstacle to the achievement of the goal. Or, in case something is accomplished by chance, that is, serendipitously, this would cast some doubts on the way in which things were originally pursued (Dunne 1993). That is, the reliability of the technique used.

As Landeweerd and colleagues (Landeweerd et al. 2015) observed, RRI is a departure from the idea that the governance of science can only be accomplished by experts, and so reducing deliberation to some form of evidence-based problem-solving (Biesta 2012). As we noted above, RRI opens up to a multitude of subjects, named “stakeholders”, that can actually contribute to the identification of the so-called grand challenges. Spruit and colleagues, for instance, posit that RRI is characterized by a shift from “assessing the desirability of the outcome of innovation processes”, often focusing on harmful product outcomes, to “assessing the qualities of the innovation process” (Spruit et al. 2015). Wilford takes a step forward in claiming that RRI creates a step-change in the way that those who are engaged in research and innovation should consider the impact of what they do (Wilford 2015, p. 348). This potentially introduces an important element that is intimately related to the phronesis based approach we are going to discuss. That is, the focus on the *rough grounds* of research and researchers’ lived experiences (Manen 1990). More specifically, the phronesis based approach precisely acknowledges the importance of addressing research as it is actually *lived* and, in doing so, it stresses its unpredictability and open-endedness, which is what characterizes virtually all approaches that take complexity seriously (Carr and Hartnett 1996; Coles 2006; Reed 1996).

As just noted, in the technocratic approach to deliberation the means to achieve a goal are worked backwards directly from the goal itself. That is where objective analysis comes into play. A rigorous objective analysis aiming at demonstration allows us to single out the relevant cause-effect relationships concerning the means so as to better guide our actions. It is worth noting, though, that the efficacy and effectiveness of means can only be fully recognised *retrospectively*. That is, after a course of action has come to an end. Conversely, by taking complexity seriously the phronesis-based approach posits that human intervention takes place in irreversible time (Snowden and Boone 2007). That is to say, we cannot travel back in time and correct our mistakes. So, unless we are dealing with situations that repeat identically over a period of time, we cannot realistically rely on the type of predictive knowledge built on generalisations from previous cases. Thus, predictive knowledge fundamentally lacks the capacity to guide the course of events as they unfold, as it is built on *retrospective generalisations*. These are generalisations formulated on the basis of the analysis of cases that have already come to an end (see (Taleb 2010) on the so called “Narrative fallacy”). Consequently, the instrumentality of means remains opaque and fundamentally hypothetical (Jullien 2004). Generalisations may have a heuristic function. Indeed, they may suggest a possible course of action. However, their validity remains tentative and similar to *guesswork*.

### The phronesis-based approach: intervention as full engagement in deliberation

In the phronesis based approach to deliberation the opacity of the means-end chain is fundamental, as it allows us to take the complexity of human intervention seriously. In the technocratic approach the relationship means-end is viewed as transparent so that goals can be achieved *directly*. The word “directly”, as Kay observed (Kay 2011), means that given a goal or an end, we can derive a technique or a procedure that allows us to achieve the goal *without roundabout means*. Conversely, in the phronesis based approach deliberation is intrinsically *oblique*, to use Kay’s terminology. Which means, first and foremost, that there is always a gap between what we may hope for, our *high-level objectives*, and the actual translations of those into the real world (Kay 2011). Means have their own autonomy and they cannot be derived so easily from an idea. Their adaptation is, in other words, always problematic (Jullien 2004, pp. 35–36).

So, in this sense, we may think that the main goals for RRI (i.e. facing the grand challenges of our time, the engagement of an informed public in identifying the societal priorities, etc.) are more like high-level objectives, ideals, rather than mere goals to accomplish like, for instance, arriving at the office on time, delivering a good presentation at a conference, or finding a time slot for a faculty board meeting. We may say that they are a direct expression of what Rawls called “the ideal theory”, which specifies *the best that we can hope for* (Rawls 1971).

The fact that the phronesis-based approach takes seriously the gap between high-level objectives and more intermediate goals means that deliberation focuses on what Chia and Holt termed indirect ways in assonance to Kay’s obliquity (Chia and Holt 2009). That is, instead of trying to find a bird’s eye view approach to take full control of otherwise open-ended processes, indirect ways make use of strategies as they emerge in due course in an extensive effort to *muddle through* (Mintzberg et al. 1976). That forbids *naive* reliance upon predictive knowledge, while it turns the attention to *immediate concerns* and corresponding activities of *practical coping* (Chia and R. and Holt 2006), which ultimately rely on the ability to *tinker with chance events* (Bardone 2016) and to work and create with *contingency* (cf. (Hyde 1998)).

Obliquity, indirect ways, tinkering with contingency, all point to an approach to deliberation characterised by the absence of a reliable technique to figure out how to proceed. Conversely, it points to the exercise of discretion and the application of judgment as an alienable attribute of the human agent, which forces us to stand “in the openness” (Hansen and Amundson 2009).

Interestingly, since deliberation resorts to discretion, the criterion of *correctness* of deliberation cannot be directly inferred from predictive knowledge. Correctness can in fact only be assessed *afterwards*. Therefore, it can never be the guiding principle for applying judgment and exercising discretion in situations as they unfold, as we have already noted above.

This has an important consequence concerning the relationship between knowledge, on the one hand, and judgment and choice, on the other. What acquires importance in the context of making a decision in the phronesis-based approach is not so much the traditional notion of expertise as the more elusive notion of sagacity (Merton and Barber 2006). As far as we are concerned here, sagacity is not to be intended as a specific type of knowledge enabling a person to gain control over a specific practical domain. Conversely, it points to the generally unassorted amalgam of skills, competences, knowledge – acquired in different times and situations – which, nonetheless, may come *in handy*, when and if circumstances allow. We claim that this amalgam of skills, competence, types of knowledge, etc., cannot really be identified or described in its totality. Nor can it be consciously developed independently from the *actual* and *concrete* situations one happens to face in one’s own profession, of which they are a direct manifestation. This means that there is nothing like a body of expertise that can be clearly pinned down beforehand, as the type of knowledge required becomes clear only in due course, that is, only as a result of being fully engaged with the particular situation at hand.

The phronetic conception of deliberation that we have briefly sketched out bears important connection with reflexivity, which we have mentioned in the introductory section as one of the RRI pillars. What has been argued by several authors (Forsberg 2015; Stilgoe et al. 2013) is that the role of reflexivity would be that of holding “a mirror up to one’s activities commitments and assumptions, being aware of the limits of knowledge and being mindful that a particular framing of an issue may not be universally held” (Stilgoe et al. 2013). This conception fairly matches with the interpretation of the term that Sandywell 1996 contributed to develop, according to which reflexivity is a fundamental component of what we may call “public rationality”, as it fosters – among others – critical interrogation of the social conditions, dialogue, historicity, socially negotiated meaning, and socially informed, critical and thoughtful action (cf. (Kinsella 2012)). In the particular context of RRI, reflexivity clearly helps bring to the fore questions related to values and beliefs, which inevitably emerge in research-related processes along with our ignorance and finitude as ontological dimensions (Groves 2009). In this sense, reflexivity goes beyond reflection, as it does not solely regard reflecting on the so-called first-oder realities. Reflexivity is in this sense what Arendt (1977) has identified with the most important aspect of being human, namely, thinking as a public good.

The phronesis-based approach is very much in line with this point of view. However it adds an important element (cf. (Hostetler 2016; Kinsella 2012)). In the light of phronesis we may argue that reflexivity is *already* embedded in the exercise and application of judgment (Birmingham 2004), which, in turn, is rooted in the researcher’s formation (*Building*) (Green 2011). In a way, we may argue that phronesis allows us to see *formation* as a key element enabling responsibility to emerge within the researcher, because it is formation, rather than reflexivity, that prepares researchers to apply judgment what concerns the role that their own work may have within the larger spectrum of social concerns and public hopes. In other words, it is the researcher’s formation allowing him or her to remain attentive to the context and to adopt a more anticipatory and responsive stance, which is central to RRI (Stilgoe et al. 2013).

Focusing on formation also rehabilitates a family of apparently pre-reflective cognitive “faculties” – often undermined because of their inevitable ambiguity and vagueness, in which a summary appraisal of a situation is already made without indulging in any process of further reflection. We are talking about abilities such as intuition, perception, acuity of vision, and ability to read the situation (Hostetler 2016; Kinsella 2012).

This last consideration leads us to pointing out that in the phronesis-based approach the interpretation that is given to intervention is not that of making or producing a certain state of affairs, but that of *active* or *full engagement* as a result of the cultivation and nurturement of the researcher’s formation. This is an important point, because it allows us to broaden up the discussion concerning what we may call “RRI-related” issues. We may then argue that *doing* RRI can be viewed as essentially rooted in the way in which researchers (and other parties concerned) practice, interpret their own work and make sense of it *for themselves*.

In the light of what we have argued, RRI may well concern and refer to research from the researcher’s point of view, that is, the point of view of *responsible professionals* (cf. (Grinbaum and Groves 2013)). There are, in fact, a number of issues and predicaments that have a larger impact on research (and innovation) than we may think. For instance, developmental issues like the formation of one’s worldview and intellectual conscience concerning science and research (Nixon 2004), emotions in the academia (Bloch 2012), competitive identities (Elizabeth and Grant 2013; Malcolm and Zukas 2009), all issues that are often neglected.

In this sense, what is central to this interpretation is the notion of human agency that casts the attention on “purposive acts of knowledgeable agents that intervene in the relevant process and that, at any point in time, could have acted otherwise” (Giddens 1979; Pandza and Ellwood 2013). The centrality of human agency implies reconsidering what responsible research may stand for, as it may refer to a variety of issues, which are rooted, and therefore inseparable from the way in which researchers and other parties concerned go about their own day-to-day activities (Gjefsen and Fisher 2014). In the technocratic approach, as we noted above, the emphasis is placed upon techniques like for instance those related to managing and assessing risks. Conversely, in the phronesis-based approach such techniques may indeed still play an important role. However, it is only the exercise of discretion and the application of human judgment in specific situations as they arise using one’s own sagacity that allows researchers along with other parties involved to take the responsibility for what they are doing.

In a way we may argue that, if we re-locate responsibility in the exercise of discretion and the application of judgment, which in turn is rooted in one’s own engagement with research and his/her character, we may broaden the spectrum of, say, RRI sensitive issues so as to involve all forms of science and types of research. That is, we do not exclusively identify RRI issues in the so-called *ethically sensitive areas*. But in the phronetic space, which is precisely the one in which researchers and other concerned parties inevitably have to tinker, apply judgment, exercise discretion, etc. Before we will come directly to discussing the meaning and interpretation given to responsibility, which we have just mentioned, we will address a preliminary question that deals with *normative commitment*.

## Normative commitment and the role of ethics

By “normative commitment” we essentially refer to the constellation of values, principles, dispositions, and strategies that come to inform and guide one’s behaviour either tacitly or explicitly. In other words, as far as we are concerned here, the term “normative commitment” regards the way in which human agents – researchers, in our case – engage with values, principles, etc. (Dreier 2002).

### The technocratic approach: applied ethics as a reflexive add-on

As we have observed above, the technocratic approach posits that the pursuit of a goal is inherently an objective, technical matter. That is, it is about bringing the relevant causal factors under one’s rational direction so as to achieve the desired outcome. What logically follows is an unbridgeable separation between what should be done in the *technical* sense and what should be done in the *ethical* sense. The former is informed by predictive knowledge, whereas the latter by ethical principles. Virtually, there are no ways in which the two camps may come to overlap.

Because of this rigid split between what is technical and what is ethical, normative commitment is essentially a source of constraints to a process that relies on strategies which are fundamentally alien to the ethical discourse. Nozick termed this way of looking at normative commitment (and ethics) “the-side-constraint” view (Nozick 1974). Grinbaum and Groves posit that according to this view ethics is essentially a source of immutable decrees, which establish what is wrong and what it right (Grinbaum and Groves 2013). This view is *deontological* in essence, and consequently the resulting conception of responsibility is related to compliance with pre-existing rules.

For example, in doing research we may say that there are technical rules pertaining to the way in which research should be conducted to achieve good results. Ethical rules are not of the same kind, as they constrain behaviour so long as ethical principles are concerned. That is, ethics comes into play, when a piece of research touches upon an *ethically sensitive area*. So, in conducting a research involving personal data, the researcher should comply with rules enforcing the protection of privacy. In this sense research and ethics meet *incidentally*. This way of thinking has been widely adopted in the various applied ethics approaches, which have flourished in the last decade or so in order to make the governance of science more ethically friendly, so to say (Landeweerd et al. 2015).

The split we have just mentioned has several conceptual consequences worth mentioning here. The first is that a class of people, namely, ethicists, is then designated as experts. Their main function is to inform researchers about the ethical constraints. Secondly, ethics is fundamentally an *add-on* (Felt 2014). That is, something externally added to the pre-existing pursuit. Which implies that there is no way to steer or influence the pursuit internally, because that would mean to interfere with the accomplishment of goals, which is inherently a technical issue, as we noted above. Thirdly, since constraints are often set on undesired impacts, the resulting ethical discourse is inevitably biased towards a vocabulary in which risk and safety are predominant, if not hegemonic, in spelling out issues and priorities – what we want. Fourthly, it follows that the areas of major concern are consequently identified among those disciplines, which fit in with the “risk discourse” (Zinn 2010). So, for example, disciplines like nanomedicine are considered more sensitive than, say, ethnology or sociology, because it is in a way easier to frame their applied outcomes in terms of the oppositional pair “undesired/desired outcomes”. That is why, we posit, the social sciences and humanities are so often viewed as playing a mere *ancillary* role (Felt 2014).

### The phronesis-based approach: the centrality of inner goods

The phronesis-based approach takes a different route. As we argued above, what is central to the phronesis-based approach is the idea that the researcher’s agency takes central stage. That is, it does not contemplate a rigid split between means and goals. Consequently it attributes a fundamental role to one’s ability to apply judgment and exercise discretion in concrete and specific situations that defy the rigid application of a certain technique (or a set of techniques). That implies that it is harder to draw a sharp line between ethical rules and principles, on the one hand, and mere technical rules, on the other, as they inevitably come *together* in the person who acts. In the phronesis-based approach, we posit, one’s normative commitment cannot be separated from one’s active and full engagement with the practice itself.

In this respect, RRI seems to represent a major step forward in comparison with other applied ethics approaches, because it places more emphasis on the process of research and innovation (Gjefsen and Fisher 2014; Oftedal 2014). We may call this “embeddedness”. Which means that to be engaged in RRI-related activities would imply to be fully part of the very process one studies. In other words, it is a call for the identification of issues as part of one’s sense-making process. And this can be done regardless of one’s disciplinary background and the outcomes that a particular discipline can have.

We may take a step further arguing that responsible research may not be viewed as exclusively pertaining to the so-called ethically sensitive areas in medicine and engineering with the social sciences and humanities acting like reflective add-ons (Felt 2014). Conversely, it may potentially identify a cluster of issues, situations, and questions – all chiefly concerning research as it is lived. In this sense one’s normative commitment cannot be reduced to, or exhausted by, the mere compliance with a set of rules or principles externally appended – what we may call the *checkmark ethics*. Therefore, one’s normative commitment can be profitably identified in the *midst* of research as a lived practice. As such, we claim, it deals with the embodiment of the so-called “internal goods” of research.

The notion of internal goods was introduced by Macintyre to go beyond a reductionist interpretation of morality centered on rules, rights and duties (MacIntyre 1984). To illustrate his point Macintyre makes the following case. Suppose that we would like to teach a child how to play chess. In order to do that, we may trick him into playing by giving him, for example, some candies every time he plays. Alternatively, we may try to engage the child *by inviting him to play chess* hoping that sooner or later he will find some interest in it *for what it is*.

According to Macintyre, the main difference between these two approaches is that in the first case we would rely on *external* goods – the candies, whereas in the second case we would try to engage the child on the basis of what he termed *internal* goods. A candy is an external good, because it can be pursued as a good (something to desire or worth pursuing) *independently* from what one is actually doing – whether he is playing chess or any other game. The same goes for power, prestige, money or fame, for instance, which, indeed, can become objects to pursue regardless of the particular practice one is engaged with. We can invariably pursue power or fame as academics, politicians, entrepreneurs, etc.

Conversely, an internal good is *always specific* to the practice we are involved in and it requires the person’s active and full engagement. As such, we can identify and recognise them if we have gained relevant experience and familiarity with the practice (MacIntyre 1984, p. 220). In other words, it implies to take the point of view of those who are working from within the practice. For an internal good is fundamentally pursued *for its own sake*.

It is worth noting that the inner goods of a practice are not inherently ethical. They cannot be mistaken for some kind of “checkmarks” to have. Nor do they refer to abstract ethical rules or principles that are somehow appended from the outside like in the-side-constraint view. Conversely, as we have just noted, they address questions related to the ends of the practice and as such they identify what is good within the practice itself, where the word “good” may refer to something that is *ideally well crafted*, done with *care* and *desirable*. In this sense the ethical dimension is *already* operating even within those aspects of the practice that may appear merely technical. Internal goods are therefore *embodied* rather then *applied*. More specifically, internal goods are embodied in those virtues enabling us to actively and profitably take part in research. Among those virtues we may find, for instance, humility and scepticism (Merton 1973), criticism (Popper 1970), flexibility and open-endedness (Kuhn 1970).

We claim that reflexivity, which we have mentioned above as one of the RRI pillars, bears an important relation with the notion of inner goods. Consider the following case.

In a study published in 2005 Gabehart showed that about 30 % of the citations 211 retracted articles obtained were after retraction (Gabehart 2005). Surprisingly, only 3 % of those citations were actually negative. This is indeed surprising, because we would expect, at least, the number of negative citations to be higher after retraction. Conversely, the fact it only occurred in a very small fraction of papers seems to suggest that post-retraction citations occurred indiscriminately well beyond the function of the so-called “ceremonial citations” (Via and Schmidle 2007). As Smith argued in commenting this very case, considerations related to impact factor may have encouraged researchers to do so (Smith 2006).

Smith adds to the list other surprising facts related to the so-called “citation behaviour”. That is, it is not surprising to see researchers erect “citation cartels” with the specific aim to artificially increase their impact factor. So, for example, the editor of a journal may ask authors to cite articles published in the same journal in order to get published. In the same vain, Matías-Guiu and García-Ramos reported about the practice of “chopping” one single research into small chunks with the very same intent to artificially increase the number of publications and therefore potential citations. It is difficult to see that more articles may lead to more citations.

As far as we are concerned here, we would not see these as examples of ethical misconduct. Unless it is clear that a researcher was actually forced to cite papers published in a certain journal, we would not see any clear misconduct. Merton reported in the past on all those biases that researchers often fall victim of (Merton 1968). That is the case, for instance, of the so-called Matthew effect, which states that it is more likely that often cited papers get more citations according to the very same principle as “the rich gets richer”.

We would regard them as examples of lack of reflexivity. That is, reflexivity would call into doubt the explicit pursuit of a good external to research. Such pursuit would in fact create friction with the internal goods of research itself and casts doubts in the public eye on the trustworthiness of research itself and its value as a social practice.

As a consequence of this lack of reflexivity, one’s engagement with research is inevitably impoverished. Whether or not the impact factor actually measures scholarity, its direct and instrumental pursuit has nothing to do, for instance, with improving the reliability of the study or the originality of one’s ideas, which can be seen as inner goods. Quite the contrary, targeting the impact factor interferes, for instance, with the actual pursuit of inner goods, as it strays from the kind of commitment to knowledge researchers are supposed to embody as men and women of science.

In general, the reference to inner goods potentially opens up to an interpretation of responsibility that is a non outcome-based conception of responsibility, namely, responsibility as care, which has been put forward by several authors in the debate around RRI (Grinbaum and Groves 2013; Stilgoe et al. 2013). More specifically, we claim that, while it is not possible to directly identify the potential pitfalls of research and innovation (see the first section of this article), a normative commitment based on the embodiment of and care for the internal goods of research, may have significance results, even when it does not directly address questions related to risk or safety.

## Responsibility

### The technocratic approach: responsibility as outcome-based

As we have argued in the first section, the technocratic approach posits that intervention is fundamentally a form of production. We intervene in the world in order to bring into existence an ideal or a model, which, therefore, precedes our engagements with the world. That is, it comes *before* we act in it and it therefore gives our activities meaning and significance. What logically follows is a conception of responsibility that is essentially *outcome*-based. That is, it is based on the achievement of a certain outcome.

If we act with the specific intent to bring about a certain state of affairs, then what turns out to be central is knowledge of causality, as Adam and Grooves pointed out (Adam and Groves 2011). When we say, for instance, that somebody is responsible for something, we tacitly assume that there is a specific causal relationship linking what a person does and the world. When this causal relationship is looked for in the past, then we speak of responsibility as *liability*. For example, if we say that Jane is responsible for the accident that has recently occurred in my block, then what we mean is we are in the position to identify the sequence of events showing Jane’s decisive role in causing the accident (Adam and Groves 2011). This conception of responsibility is used in court. As far as the technocratic approach is concerned, there is another form of outcome-based responsibility that is worth discussing. That is, the one ascribing responsibility to an individual (or a group of individuals) for a future event. In this case the causal relationship between what we do and the world is actually located *in the future* in terms of what we would like to achieve. That is, a causal relationship exists, not for a past event like in the case of liability, but it is assumed for a future one. That is in a nutshell the notion of responsibility as *accountability* (Giri 2000), which is very much attached to the deontological approach we referred to above (Grinbaum and Groves 2013).

Since the technocratic approach assumes that an intervention aims to produce a certain outcome, then the ascription of responsibility is preordained to taking upon one’s shoulder the task to *produce* (in the sense of bringing into existence) the desired outcome. In this sense responsible research can be interpreted as the one in which researchers (and other parties concerned) are held *accountable* for bringing about what is desired or/and for avoiding what it is not desired. It is only then that we may say that he/she is actually *responsible*.

Worth noting, as responsibility is linked to knowledge of causality, the outcome, which one should produce and therefore he/she is held accountable for, should be known in advance.

Grinbaum and Groves rightly pointed out that the whole idea of responsible research and innovation is characterized by some conceptual and practical difficulties, because we are essentially dealing with something that is in a fundamental way *future-oriented* (Grinbaum and Groves 2013). Both research and innovation are essentially affected by uncertainty and indeterminacy. As they put it, “being responsible becomes subject to increasing uncertainty” (Grinbaum and Groves 2013, p. 122). In this sense the notion of responsibility that we are looking for inevitably has to have a “prospective dimension” (Owen et al. 2012, p.31). It follows a fundamental disaffection with approaches that focus on accountability, liability and (causal) evidence (cf. (Stilgoe et al. 2013)), which contribute to a general re-conceptualization of the term “responsibility” as care, which is the main topic of the present section.

### The phronesis-based approach: responsibility as care

As just noted, the open-ended character of research and innovation makes it virtually impossible to ascribe responsibility to an unknown event in this specific sense. It reduces the space of action to the achievement of an outcome that is *already* known. Whereas, as Grinbaum and Groves brilliantly pointed out, “creative action and innovation point forwards, opening up the world the past has created and adding new entities to it that change the way it works” (p. 124). In doing so a conception of responsibility as accountability restricts the way we can actually express our human agency and creativity, which is central to the approach based on phronesis, as we have noted in the previous section. Besides, outside of a narrow deontological interpretation of our ethical commitments, the whole notion of responsibility can be viewed not as something that is *imputed* to us (as it is for accountability), but rather as something that we *actively take* (Adam and Groves 2011). That is why, if we move away from the technocratic approach, another, strictly *non* outcome-based and *future-oriented* interpretation of responsibility becomes possible (cf. (Grinbaum and Groves 2013)). And that is the idea of *responsibility as care*.

Consider, for example, the case in which a university professor feels responsible for her PhD students. If by that we mean that she is accountable, then we should specify the outcome she is actually held responsible for. That is, the one that she has to produce. This outcome should be indeed *known beforehand* and it could be, for example, the number of students successfully defending their PhD in five years. She would be then held responsible for the PhD student, because she has to *hit the numbers*.

However, at least on a mere intuitive level, we feel that there is something more. The word “responsibility” may refer to the fact that the supervisor in question is responsible in the sense that she actually *cares* about her PhD students and is *responsive*.

Care and responsiveness refer to the fact that responsibility is always related to “responding to somebody”, to “giving an answer”. But not in the narrow sense of providing an account of why one has been doing this or that, as Lucas (1993) argued. We are talking more about a negative form of responsibility, which, according to Lucas, is not pressing for an account, but pointing to a duty of care, which is the kind of response that we own “in the here-and-now of praxis”, as he put it (Lucas 1993, p. 53).

More in general, we may argue that care in this case is a type of engagement with the world (Heidegger 2010), which takes a different perspective than responsibility as providing an account justifying one’s action (often *a posteriori*). First of all, care is an act that is done for its own sake. That is, the supervisor would care about her students regardless of *hitting the numbers*. Which means that care, strictly speaking, has *no instrumental value*. That is, it is not imputed in relation to a goal to achieve. Conversely, we argue that care is rooted in one’s own agency, as it presupposes the type of engagement in which a person is called to exercising discretion and apply judgment, as we noted above.

Besides, it also reflects the way a person is *as a whole* and so comprising her own values, habits, competences, knowledge, namely, her *entire worldview*. In this sense care does not, and cannot refer to a legalistic framework based on the compliance with a set of rules of conduct. The notion of care reaches beyond, as it appoints the individual with the power to take initiative and act in the world.

It follows that care is not just a moral disposition exhibited by good-hearted people, as it holds on to the way in which a person *makes sense* of her engagements with the world. To go back to the example, a supervisor cares for her PhD students in a sense that her acts and deeds – what she does and says – are the fullest expression of her way of going about her profession, her making sense of it.

There is an additional element that is worth mentioning here. As Gilligan famously argued, care does not involve a commitment towards the world in the abstract sense of universal rules of conduct (Gilligan 1982). Conversely, the act of caring is always attuned to the everyday *ongoings*, that is, the *here-and-nows*, which connect us back to exercising discretion and applying judgment, as there are no techniques or recipes that inform directly the way we should act.

More generally, care is not limited to the taking care of another human being. It is the care of one’s own disciplines and the possibility to advance in knowledge. As such, care is an expression of something coming out of oneself, as just noted. It follows that the interpretation of responsibility as care depicts the researcher, to use Gadamer’s words, “directly confronted with what he sees” instead of “standing over against a situation that he merely observes” (Gadamer 2004, p. 324). This element connects back to the idea of intervention as engagement we discussed in the first section.

So, we may now argue that responsibility as care refers to a kind of *day-by-day* type of *intervention* related to the praxis (Lucas 1993), which is not exclusively moral, as it gives up on any *deontological* commitment to bringing about a certain *known* state of affairs (as opposed to responsibility as accountability), while remaining open to what can actually be achieved in the specific context of one’s action.

One last consideration. As we have argued before, the phronesis based approach supports the so-called oblique or indirect ways. That is, when we care, we are engaged in the world not to fulfil a goal. But we are immediately attuned to, and therefore taking care of the rough grounds of the practice, the day-to-day activities, while renouncing to address directly what we may call “the grand challenges”, our high-level objectives. Yet we claim that taking care may help get closer to the same very high-level objectives but indirectly. Interestingly, this is well formulated in the British proverb “take care of the pennies, and the pounds will take care of themselves”. More specifically, we may see care as that which tries to bridge the gap between our high objectives and the more mundane level of our day-to-day activities.

## Parting thoughts. Towards a phronetic space for responsible research (and innovation)

What we have tried to sketch in this paper is an approach based on the Aristotelian notion of phronesis, which may help explore the plurality of issues that may go under the general label of “responsible research”. In turn, this exercise, which is eminently conceptual, also aimed to broaden up the current discussion related to Responsible Research and Innovation (RRI), which seems to stagnate around various techniques of risk management and procedures of public participation.

Specifically, we have tried to argue for the importance of *re-locating* responsible research and innovation into a phronetic space. Such phronetic space is not meant to identify techniques to apply or ethical codes to follow. Conversely, it focuses on the idea of responsible research as the cultivation and nurturement of the researcher’s formation as a type of engagement with the actual practice of research for what it is – an open-ended enterprise. That is to say, an enterprise that is mainly devoted to exploration, inquiry, and discovery *done for its own sake*.

As we pointed out, if we acknowledge the fundamental open-endedness of research as a practice, responsible research cannot be something one is simply *imputed*. Conversely, it is something that is *taken* and so *embodied* in the researcher’s agency and that of other parties participating in the world of research directly or indirectly. Such agency is exhibited in intervening in relevant processes that, at any point in time, could be *otherwise*.

As we have tried to show, this means, more specifically, that intervention, and consequently deliberation concerning how to proceed, cannot be reduced to a technique or formal procedure informing the *making* of something. Conversely, it is essentially rooted in the researcher’s exercise of discretion and application of judgment, which cannot be categorised resorting to expert judgment. It is an expression of one’s own active and full engagement.

The exercise of discretion and judgment along with the development of sagacity is not arbitrary, a-moral, aprioristic or free from any normative considerations concerning how one should act. That is because the practice has its own internal goods pointing to what is desired, done with care and well crafted, which researchers (and other parties concerned) come into contact with by experiencing and living research for themselves (Reed 1996), not by rigid compliance with some kind of ethical code. This is a fundamental step to take, for example, towards seeing the alienation of research from researchers as a major source of irresponsibility. By that we mean those external interferences diverting researchers from full engagement and the embodiment of the internal goods. That includes, indeed, private interests, but also 1) aggressive policies enforcing accountability favouring conservative tactics rather than promoting the pursuit of new directions (Shore and Wright 2015); 2) the discourse around the *impacts* (Briggle 2014; Watermeyer 2014) and their effect on academic identities (Watermeyer 2015); 3) the shortsighted views on indicators of scientific productivity and excellence in academia (Wood 2012) that, once indiscriminately targeted for their own sake, lose their own reliability (Foster et al. 2015) and, at the same time, contribute to the emergence of epistemic inequality among the different disciplines and scientific cultures (Lõhkivi et al. 2013).

Re-locating responsibility into the researcher’s phronetic space means that responsible research is ultimately a form of taking care, which already points to a relational aspect implicit in one’s work that reaches far beyond the discourse around public participation and engagement. Here care refers primarily to the way a researcher (and other parties concerned) relates to people he/she gets in touch with as well as to research as a public good. The idea of research as public good goes well beyond the instrumental and reductionist view of research and science, according to which one’s contribution is assessed in terms of the impacts – real or merely imagined – that it might have on the GDP. Conversely, research as public good is related to the idea that research is an expression of what Karl Jaspers in his *The Idea of University* (Jaspers 1959) called “man’s fundamental and primary thirst for knowledge”, which, as such, proceeds, although it is not in contrast with, all considerations related to usefulness. Such a thirst for knowledge goes well beyond simple curiosity, and it can be viewed as an enduring (and sometimes tantalising) interest that views exploration and inquiry as a way to establish a contact with the world outside oneself, as Polanyi put it (Polanyi 1964).

It is worth noting that the type of engagement that we have described is not to be taken as a normative model to force upon researchers and all other parties concerned. Conversely, I posit that it should be viewed chiefly as an educational challenge for research and teaching institutions, single researchers and the society as a whole. By “educational challenge” I mean that responsible research is essentially a type of engagement to develop in time rather than an outcome to bring about once and for all. That is, it is located in, and is not separable from, one’s own way of becoming a part of the “practice” of research for oneself.
